# Moderators of Perceived Effort in Adolescent Rowers During a Graded Exercise Test

**DOI:** 10.3390/ijerph17218063

**Published:** 2020-11-02

**Authors:** Gerd Schmitz

**Affiliations:** Institute of Sports Science, Leibniz University Hannover, 30167 Hannover, Germany; gerd.schmitz@sportwiss.uni-hannover.de; Tel.: +49-511-762-2191

**Keywords:** adolescence, perceived exertion, cognition, mental health, rowing

## Abstract

Physical activity promotes mental health. A key factor is self-regulation. In the field of sports, self-regulation is related to the psychophysiological competence of rating of perceived effort (RPE). It was reported that adolescents have lower RPE competencies than adults, and it was hypothesized that this effect depends on physiological and cognitive development. The present study investigated in a sample of adolescents whether the RPE is related to basic cognitive competencies. Twelve rowers performed cognitive tests and a graded exercise test on a rowing ergometer, in which they continuously rated their perceived effort. Objective load measures and subjective perceptions were highly correlated (rho = 0.95–0.99). Furthermore, these correlations were inter-individually moderated by measures of mental speed and spontaneous flexibility. The results confirm the significance of basal cognitive competencies for conscious load perception. It is discussed whether regular sport has beneficial effects on the development of RPE competencies by enhancing cognitive regulation.

## 1. Introduction

Physical activity promotes the mental health of adolescents [1]. Adherence to physical activity is a great individual challenge [2], and the key factors are motor competences, control and self-regulation competences as well as perceptual competences [3,4]. In the field of sports, these competences refer to the ability to plan, execute and regulate physical performance autonomously and to choose adequate loads for achieving physical and mental health. A significant perceptual component is the competence for rating of perceived effort (RPE). Accordingly, RPE-based load regulation is a widely applied method in health sports [5,6]. Investigating the factors of the RPE is relevant for evidence-based health promotion through improvement of self-perception, effective self-regulation as well as for supporting personality development [5,7]. The present study investigates the relationship between RPE, cognitive information processing speed and cognitive flexibility. 

RPE is regarded as psychophysical competence [8], which develops during maturation [9]: Until the age of 12 years, children are only able to discriminate up to four intensities. During adolescence (13–18 years) a more fine-grained perception develops, and adolescents become able to differentiate 10 to 15 intensity levels [9]. However, the RPE performance is still lower than in adults and the reasons are still unclear. Groslambert and Mahon [9] hypothesized that cardiorespiratory factors become more important for the RPE with increasing age. Furthermore, as the RPE also has cognitive components, it seems to depend on the cognitive developmental status. Physical activity typically declines in adolescence [10]. Investigating the mediating factors of the RPE is important for understanding control and self-regulation as well as engagement of adolescents in physical activities.

### Competence for Rating of Perceived Effort

The RPE results from the integration of multiple afferent signals from the body periphery into a single percept. It is a subjective measure, but the rating of perceived effort correlates highly with objective cardiorespiratory and metabolic parameters. This has been shown across nearly all age groups and in a variety of populations: trained, untrained, sedentary, overweight persons and coronary heart disease patients [11,12,13,14]. 

St.Clair-Gibson [6] understood the perception of effort as an exchange of information between the brain and the peripheral physiological system involved in energetic regulation. Furthermore, according to the Corollary Discharge hypothesis, the brain makes assumptions about how the effort will feel in the near future based on motor commands to the muscles [15]. The underlying concepts correspond essentially to the idea of forward modelling in motor control. Here, the anticipated feeling of the movement has direct consequences on perception and action [16]. 

Empirical findings from studies with adults highlight the significance of specific cognitive abilities for RPE. Lohse and Sherwood [17] showed that the attentional focus affects RPE. Schmitz et al. [18] investigated individuals with and without intellectual disabilities and found weaker correlations between heart rate and RPE in individuals with intellectual disabilities. Furthermore, they reported significant correlations between RPE and the speed of information processing as well as cognitive flexibility in male soccer players. In a follow-up study, Schmitz and Sommer [19] replicated these results with female soccer players. Anderson et al. [20] assign these abilities to the executive control system of children, which is a framework derived from developmental neuropsychological literature. It subsumes information processing (including processing speed), cognitive flexibility, attentional control and goal setting as main components. The present study investigates the significance of the first two abilities for the RPE competence in adolescents. 

In this regard, subjective load perceptions were correlated with objective performance indicators and it was analyzed whether information processing and cognitive flexibility moderate these correlations. Information processing was operationalized by the speed of the information processing [20]. Cognitive flexibility was operationalized by spontaneous and reactive flexibility [21].

In contrast to the field study from Schmitz et al. [18], the present study was performed as a laboratory study in order to control the load and reduce the inter-individual variability. Load can be well controlled during graded exercise tests performed on a treadmill, a cycling ergometer or a rowing ergometer. The study focused on ergometer rowing, because the force production depends more on whole body movements (arm, upper body and leg movements) than during cycling and running, which might support the perception of effort. 

The study investigated the following hypotheses in a sample of adolescent rowers:

1. The correlation between heart rate and RPE is moderated by the speed of the information processing measured by the Number Connection Test.
2. The correlation between heart rate and RPE is moderated by spontaneous and reactive flexibility measured by the Five-Point Test.

## 2. Materials and Methods

Twelve junior athletes (9 boys, 3 girls) participated in the study. They belonged to a regional selection team and therefore were regarded as skilled. They were on average 15.9 years old (standard deviation SD: 1.2 years) and had 9.9 (SD: 1.1) years of education. All of them had regularly participated in competitive rowing sports. As part of their admission to the regional rowing federation, all of them had been medically examined within the last year with regard to their fitness for competitive sports. Moreover, they all answered a questionnaire from the German Society for Sports Medicine and Prevention, which is an adapted version of the Physical Activity Readiness Questionnaire (PAR-Q) of the Canadian Society for Exercise Physiology. The questionnaire yielded no results pointing to a health restriction. The participants and their legal representatives gave their written informed consent to participation. The study was performed in accordance to the Declaration of Helsinki inclusive its later amendments and had been pre-approved by the Ethics Committee of the author’s former institution, the Carl von Ossietzky University of Oldenburg.

### 2.1. Procedure

The participants were instructed to eat a sufficiently high carbohydrate diet on the test day and to take their last meal two hours before the start of the test. On the test day, each participant first performed the cognitive tests and then the physical exercise.

#### 2.1.1. Cognitive Assessment

The present study applied the same cognitive performance tests as [18,19]. Two non-verbal neuropsychological paper–pencil tests assessed the speed of information processing and cognitive flexibility.

In the Number Connection Test the participants had to connect the numbers one to ninety in ascending order as quickly as possible. On a DIN A4 sheet of paper, consecutive numbers were located directly above, below, to the left, to the right or at a diagonal position to each other. Performance time was regarded as a measure for speed of information processing. The outcome correlates on a medium-to-large level (r = 0.40–0.83) with the outcome of the intelligence tests. It is related to fluid intelligence [22,23]. 

In the Five-Point Test, the participants received two sheets of paper (DIN A4) with forty preprinted rectangles. Each rectangle contained five dots. The participants had to produce unique designs by connecting two to five dots. The instruction was to produce as much different designs as possible within 3 min while avoiding repetitions [24]. The test measured two aspects of flexibility. The number of unique designs reflects spontaneous flexibility; the percentage of perseveration errors reflects cognitive flexibility emerging from inhibition, i.e., reactive flexibility [21,25,26,27]. The normative data of adults were published by Goebel et al. [25] and Cattelani et al. [28]. A correction index for age and education has been provided by Cattelani et al. [28], allowing comparing the number of unique designs with age-standardized normative data. The correction is not necessary for perseveration. 

#### 2.1.2. Exercise Performance test

All participants were familiar with the graded exercise test, since they had already participated in a similar test one year before. All tests were performed on a Concept II (model D) rowing ergometer. The drag factor was set to 145 for men and 135 for women. A higher drag factor reflects a higher air resistance of the flywheel. One to two weeks prior to the study, all participants had participated in a 2000-max test during performance assessment in the regional selection team, in which they had to row the distance of 2000 m as fast as possible. This test is one of the standard procedures in rowing-specific performance diagnostics and is typically used by the head coach to determine the peak power (Ppeak) of each athlete [29,30,31,32]. For the graded exercise test, six submaximal intensities were defined relative to Ppeak. 

After resting for four minutes on the ergometer, the participants rowed with 35%, 45%, 55%, 65%, 75% and 85% of Ppeak for four minutes each. Before the intensity increased, they had a break of one minute. During rest, during the breaks as well as 150 s and 330 s after test termination, capillary blood samples were drawn from a hyperemised earlobe into 10 μL end-to-end capillaries to analyze the lactate concentrations. Lactate concentrations were determined by the Super GL compact system (Dr. Müller Geraetebau GmbH, Freital, Germany). The software Winlactat 4.6.2.15 (mesics, GmbH, Muenster, Germany) was used to determine the individual lactate thresholds. The lactate threshold (LT) and the individual anaerobic lactate threshold (IAT) were calculated according to the method proposed by Dickhut et al. [33]. The times of LT and IAT were determined by a polynomial fit (quartic polynomials).

Peak heart rate (HRpeak) was calculated according to the formula proposed by Hottenrott et al. [34]. Heart rate was measured continuously with 1 Hz (acentas GmbH, Hoegertshausen, Germany). The participants wore a breast belt, which transmitted the data wirelessly to a laptop computer. The software enables setting markers, which was used to denote the points in time when perceived effort was rated. Perceived effort was rated with the Borg scale. The rating scale differentiates 15 intensity levels ranging from 6 (no effort at all) to 20 (maximal exertion) [8]. Perceived effort was rated during rest, and at 60 s and 210 s of each stage of the graded exercise test. Thus, the RPE was assessed every 150 s.

### 2.2. Statistics

Means and standard deviations (SD) were calculated for the physiological data. Medians and interquartile ranges (IQR) were calculated for the data from the cognitive performance tests. Differences between the cognitive data with reference data from normative samples were tested for significance with Wilcoxon rank-sum tests. To get an indicator for the individual RPE competence, for each participant a Spearman’s rho was calculated by correlating the ranks of the heart-rate data and the ranks of the RPE data. Spearman rank correlation analyses focus on the monotonicity between two variables and can be used to analyze linear as well as non-linear relationships. To test Hypothesis 1, the rho-coefficients from these analyses were taken as the dependent variable in another Spearman rank correlation analysis and the performance time in the Number Connection Test as the independent variable. The same procedure was applied for testing Hypothesis 2 with unique designs and perseveration errors from the Five-Point Test as independent variables. The *p*-levels from the analysis for Hypothesis 2 were corrected according to the stepwise Bonferroni–Holm procedure, because the Five-Point Test provides two measures for flexibility. Furthermore, a Bayesian linear regression analysis was performed with all cognitive variables as predictors. Fisher’s z-transformation was applied to the coefficients of the Spearman rank correlations between heart rate and RPE, which were then taken as a criterion variable. Bayesian approaches have been recommended for sport scientific studies with low sample sizes [35]. Power (ß) is described for insignificant results.

## 3. Results

### 3.1. Graded Exercise Test

The mean Ppeak was 273.80 (SD: 49.03) W. In the graded exercise test, the participants yielded a maximum power output of 222.33 (SD: 46.44) W, which corresponds to 81% of Ppeak. The relative power output was 3.09 (SD: 0.69) W/kg.

**Figure 1:**
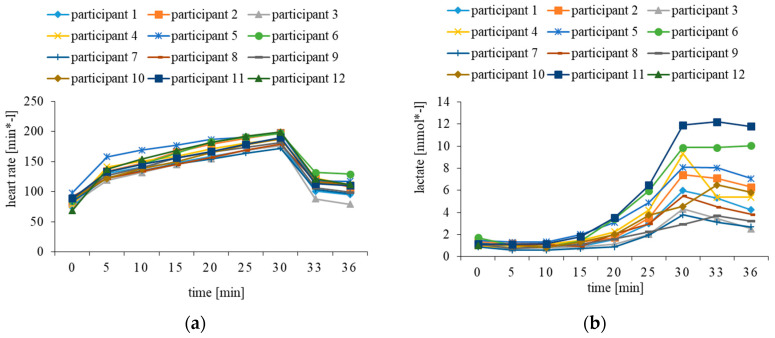
Heart rate (**a**) and lactate concentrations (**b**) before, during and 3 to 6 min after the graded exercise test.

The individual heart rate data and lactate concentrations are shown in Figure 1. During the graded exercise test, the heart rates increased linearly. The participants had a maximum heart rate of 187.8 (SD: 9.9) beats per minute. Participants 2, 5, 6 and 12 reached their HRpeak. Three to six minutes after completion, the heart rates decreased towards the initial level during rest, but none of the participants reached resting heart rate.

Lactate concentrations are shown in Figure 1b. They increased exponentially during the graded exercise test. The maximum lactate averaged 7.03 (SD: 2.6) mmol/L. The mean lactate threshold was determined at 0.93 (SD: 0.24) mmol/L and the individual anaerobic threshold at 2.30 (SD: 0.34) mmol/L. The participants reached the IAT on average after 21.5 (SD: 3.4) min. The span ranged from 17.3 to 26.2 min, indicating that the energy metabolism different inter-individually, which is also reflected by different rates of lactate decrease during the six minutes after the test had ended. 

**Figure 2:**
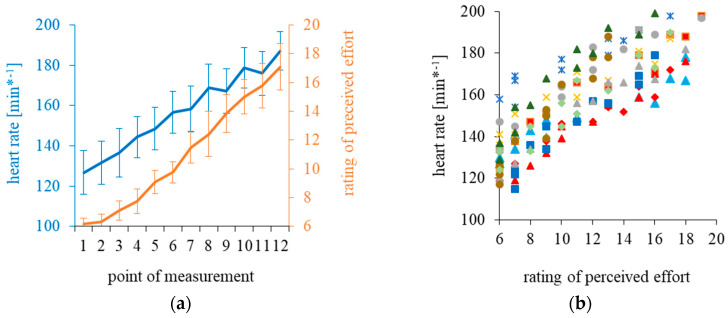
Inter-individual means and standard deviations for heart rate and the ratings of perceived effort (**a**). Individual data are shown in (**b**).

Ratings of perceived effort are plotted against the heart rate data in Figure 2. Individual data are provided as Supplementary Material (Data_Rowing.xlsx). The RPE also increased linearly during the graded exercise test. The individual coefficients of the correlations between heart rate and RPE ranged from 0.95 to 0.99 (median rho: 0.98 IQR: 0.02) and were all highly significant (*p* < 0.001).

### 3.2. Cognitive Performance Tests

All measures related to cognition were independent from age and education. In the number connection test, the participants achieved a standardized value of 103 (IQR: 15.75), which was not significantly different from the average from the normative sample (100, W = 40.50, *p* = 0.408, ß = 0.09). In the Five-Point Test, the participants produced 40.50 (IQR: 11.50) unique designs. This value was not significantly different from the mean of the normative sample (37.24, W = 50.00, *p* = 0.388, ß = 0.14). Adjusting the data with respect to age and education by the formula suggested by Cattelani et al. [28] yielded a median of 48.79 (IQR: 12.19), which was significantly higher than the mean from the normative sample (W = 75.00, *p* = 0.005). The participants produced in 2.79% (IQR: 6.25) perseverations. This value was significantly different from the mean of the normative sample (7.59 %, W = 10.00, *p* = 0.022).

### 3.3. RPE Competence

**Figure 3:**
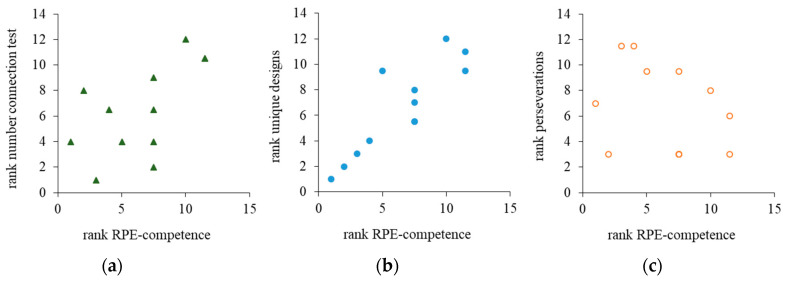
Rank correlations between rating of perceived effort (RPE) competence, performance time in the Number Connection Test (**a**), unique designs (**b**) and perseveration errors (**c**) in the Five-Point Test.

In the next step, the cognitive performance measures were correlated with the indicators for RPE competence (Figure 3). Hereto, the Spearman rank correlation coefficients from the previous analysis were taken as the dependent variable. These analyses yielded significance for the performance in the Number Connection Test (rho = 0.61, *p* = 0.035) and for unique designs (rho = 0.87, *p* < 0.001) but not for perseveration from the Five-Point Test (rho = -0.24, *p* = 0.452, ß = 0.12). Taken into account that the energy metabolism during the graded exercise test differed between the participants (Figure 1), the analysis was replicated and limited to the data recorded before the participants reached their IAT. The results changed only marginally: Spearman rank correlations yielded significance for the Number Connection Test (rho = 0.62, *p* = 0.033) and for unique designs (rho = 0.85, *p* < 0.001), but not for perseveration (rho = -0.28, *p* = 0.383, ß = 0.15).

Finally, a Bayesian linear regression analysis was performed with z-transformed RPE competence as the criterion variable and the three measures from the cognitive tests as predictor variables. The Bayes factor (*BF*10 = 38.45, R^2^ = 0.79, *p* = 0.001) was largest for a model including the number of unique designs and the performance in the number connection tests as predictors, i.e., the data were 38.45 times more likely under this model than under the null-model. The second largest Bayes factor was achieved for a model with unique designs as the single predictor (*BF*10 = 35.67, R^2^ = 0.70, *p* < 0.001). The Bayes inclusion factors for the cognitive variables were 6.40 for the number of unique designs, 0.60 for the performance in the Number Connection Test and 0.55 for perseveration. 

## 4. Discussion

Previous studies have shown that the competence for rating of perceived effort depends on the developmental status and is related to the cognitive abilities of adults [9,18]. The present study investigated whether speed of cognitive information processing and flexibility moderate the correlation between heart rate and RPE in adolescents. Hereto, the participants performed two paper–pencil tests and subsequently participated in a submaximal graded exercise test, during which they continuously rated their perceived exertion. Correlation and regression analyses show significant effects for the performance in the Number Connection Test as well as the number of unique designs from the Five-Point Test.

High correlations between RPE and heart rate indicate that the participants were very familiar with the applied method for rating of perceived effort. Furthermore, they reflect high RPE competences. Typically, adolescents show weaker correlations between heart rate and RPE. Eston and Williams [36] applied the Borg scale during a submaximal cycle ergometer test and reported a mean correlation coefficient of 0.74 for participants aged 16.0 years. Pfeiffer et al. [37] and Rodriguez et al. [38] reported even lower coefficients of 0.64 and 0.70. Thus, they suggested to use rating scales with fewer intensity categories in children and adolescents. When using such scales, the correlations between RPE and heart rate increase [39]. Nevertheless, the demands regarding the differentiation of intensities are comparatively lower than with the Borg scale. Groslambert et al. [9] argued that adolescents have lower RPE competences than adults, because cardiorespiratory factors involved in RPE might increase with age and adolescents have nearly completed their cognitive development. Adult rowers reach correlation coefficients of 0.95 with the Borg scale, which corresponds well to the mean correlation coefficient of 0.98 from the present study [40]. Thus, the RPE competence of the participants from the present study might have been fully developed already. They all were competition-experienced, probably indicating that participation in intensive sports has an accelerating effect on the development of RPE. This effect might be moderated by cognition. The hypothesis that acceleration concerns the cognitive development [9] is supported by the findings of relative higher cognitive performances of the participants from the present study compared to normative data. 

The main goal of the present study was to analyze the relationship between the competence for rating perceived effort and information processing as well as cognitive executive control. The results show that mental speed as well as spontaneous flexibility predict how well RPE correlates with heart rate. This can be interpreted in terms of a moderating influence on how heart rate, a measure for the actual effort of the cardiovascular system, is integrated into RPE. The results confirm Hypothesis 1. Hypothesis 2 is only partially supported by the results, since only one of the two flexibility components was a significant predictor for RPE competence. A moderating effect of perseveration errors on the correlations between RPE and heart rate could not be proven. The effect size of this correlation was medium, which corresponds to the reported effect size from Schmitz et al. [18]. Therefore, in the present study, the statistical power was too low to achieve a significant effect for perseveration. Furthermore, it might be considered that the participants from Schmitz et al. [18] made much more perseveration errors than the participants of the present study. As a consequence, a replication with a higher sample size is necessary to make a reliable conclusion regarding the role of perseveration on RPE in adolescents. 

The Bayesian regression analysis provides information on how likely the data occur under regression models with all variables as predictor (Bayes factor) and how likely the data occur with models that contain a specific predictor (Bayes inclusion factor). The largest Bayes factor was achieved under a model with the predictors’ unique designs and the performance in the Number Connection Test, which complies with the results of the Spearman rank correlation analyses. However, this model was only marginally more likely compared to a model with unique designs as the single predictor. Thus, spontaneous flexibility played a predominant role in the regression models on RPE competence. This is also reflected by the Bayes inclusion factor. 

The results of this laboratory study confirm those of former field studies with male and female participants performing soccer training [18,19]. They can be embedded into competence-oriented health concepts for adolescents. Stodden et al. [41] described the emerging interactions between physical activity, fitness, motor competences and perceptions of motor competences during the developmental time. Their understanding of the perceptions of motor competences includes the perception of effort (ibidem, p. 295), which is the topic of the present study. The authors hypothesized that the perceptions of motor competences depend on the cognitive development and influence how children persist and engage in physical activities. The correlations between the cognitive control and RPE in adolescents reported in the present study and in a former study for individuals with intellectual disability [18] support this view. 

Thiel et al. [5] see the perception of effort as an important competence for health in sports and exercise therapy and as a component of a movement-related control competency. Carl et al. [3] (p. 689) regard this control competence as necessary for load regulation and structuring activities autonomously in order to improve physical and mental well-being. Within their framework, it is one of three major competences enabling individuals to become “physical activity-related health competent” (PAHCO). The PAHCO-framework combines physiological and behavioral competences for a healthy lifestyle and highlights active engagement and the role of information processing capabilities. Recently, Haible et al. [42] showed that the control competences of adolescents are also associated with health- and fitness-related motivation as well as physical fitness. The results of the present study provide more insights into the control competence by revealing its cognitive components: information processing abilities and cognitive flexibility. They are two of four domains in the framework for executive cognitive control proposed by Anderson et al. [20]. Schmitz and Sommer [19] found a correlation between the RPE and a behavioral regulation index (BRI), which was orthogonal, i.e., independent from the correlation with cognitive information processing. In contrast to the cognitive tests that measure peak performances, the BRI measures the ability to control one’s own behavior and emotional processes in an everyday environment [43]. These are different and mutual amending views on cognitive/behavioral control. In the study from Schmitz and Sommer [19], the data were recorded during a one-hour soccer training, which put high demands on autonomous load regulation. Therefore, this RPE competence seems to be related to self-regulation competences, at least when physical activities require autonomous load regulation. Notably, the PAHCO framework also differentiates between the control competence and self-regulation competence, whereby the latter refers to the planning and execution of physical activities. Thus, RPE might address several health-related competences concurrently. 

Carl et al. [3] suggest not to train the sub-competencies individually, but to train also their interactions for health promotion. Hereto, interventions focusing on autonomous physical training in combination with explicit ratings of perceived exertion and pacing might already represent a basic approach. There are now reliable empirical findings on the role of cognitive information processing (speed and control) for RPE as well as indications of a relationship between RPE and self-regulation from a former study. Thus, RPE-based training seems to address several significant health competences concurrently.

## 5. Conclusions

The correlations between RPE and heart rate found in the present study were higher than previously reported for adolescents and similar to those reported for adults [9,40]. Since all participants were competition-experienced rowers, it is tempting to conclude that rowing training has an accelerating effect on the development of the perception of effort. RPE was moderated by cognitive variables. Higher mental speed as well as better spontaneous flexibility correlated with a higher RPE competence. Performance in one cognitive test was significantly better in the study sample compared to the normative sample. Whether rowing training causes these effects or whether higher cognitive performance allows a better estimation of the own state of effort should be investigated in future studies.

RPE seems to be a complex psychophysiological competence that is composed of several sub-components: afferent perceptions on effort as well as cognitive processes related to prediction and executive cognitive control. Cognitive components are the speed of information processing, cognitive flexibility and behavioral self-regulation. Whether these cognitive variables influence the integration of multiple sensations from the body periphery during sports, whether they support the prediction of future states of exertion or whether they are influenced by regular sport training and autonomous load regulation might be investigated in future studies.

## Supplementary Materials

The following are available online at https://www.mdpi.com/1660-4601/17/21/8063/s1: Data_Rowing.xlsx.
